# Studies on the binding of nitrogenous bases to protoporphyrin IX iron(II) in aqueous solution at high pH values

**DOI:** 10.1007/s00775-022-01929-4

**Published:** 2022-03-02

**Authors:** Jack Silver, Golzar Al-Jaff, Michael T. Wilson, Daniel den Engelsen, George R. Fern, Terry G. Ireland

**Affiliations:** 1 College of Engineering, Design and Physical Sciences, School of Engineering, Wolfson Centre for Materials Processing, Brunel University London, Kingston Lane, Uxbridge, UB8 3PH Middlesex UK; 2 Present Address: School of Life Sciences, University of Essex, Wivenhoe Park, Colchester, CO4 3SQ Essex UK; 3 Department of Chemistry, College of Education, Salahaddin University-Erbil, Erbil, Iraq

**Keywords:** Protoporphyrin IX iron(II), Mossbauer spectroscopy, Nitrogenous ligands, Stability constants, Hill coefficients

## Abstract

Studies are reported on the formation of low-spin six-coordinate [Fe(PPIX)L_2_] complexes from iron(II) protoporphyrin where L is one of a series of nitrogenous ligands (aliphatic, aromatic or heterocyclic). The bonding constants have been determined by titration of the metal complex with these ligands and are compared in relation to previous studies. The adduct formation was monitored utilising optical spectroscopy. In addition, Mӧssbauer spectroscopic experiments were conducted to monitor the electronic environment around the central iron atom in these complexes. The two complementary spectroscopic methods indicated that all nitrogen ligands formed low-spin octahedral complexes. The magnitude of the overall binding constants (*β*_2_ values) are discussed and related to (a) the p*K*_a_ values of the free ligands and (b) the Mössbauer parameter Δ*E*_Q_, which represents the quadrupole splitting of the haem iron. The *β*_2_ and Δ*E*_Q_ values are also discussed in terms of the structure of the ligand. Cooperative binding was observed for nearly all the ligands with Hill coefficients close to 2 for iron(II) protoporphyrin; one of these ligands displayed a much greater affinity than any we previously studied, and this was a direct consequence of the structure of the ligand. Overall conclusions on these and previous studies are drawn in terms of aliphatic ligands versus aromatic ring structures and the absence or presence of sterically hindered nitrogen atoms. The implications of the work for the greater understanding of haem proteins in general and in particular how the nitrogenous ligand binding results are relevant to and aid the understanding of the binding of inhibitor molecules to the cytochrome P_450_ mono-oxygenases (for therapeutic purposes) are also discussed.

**Graphical abstract:**

Changes in the electronic absorption spectra of five-coordinate [Fe(II)(PPIX)(2-MeIm)] that occurred as the temperature was lowered from room temperature to 78° K

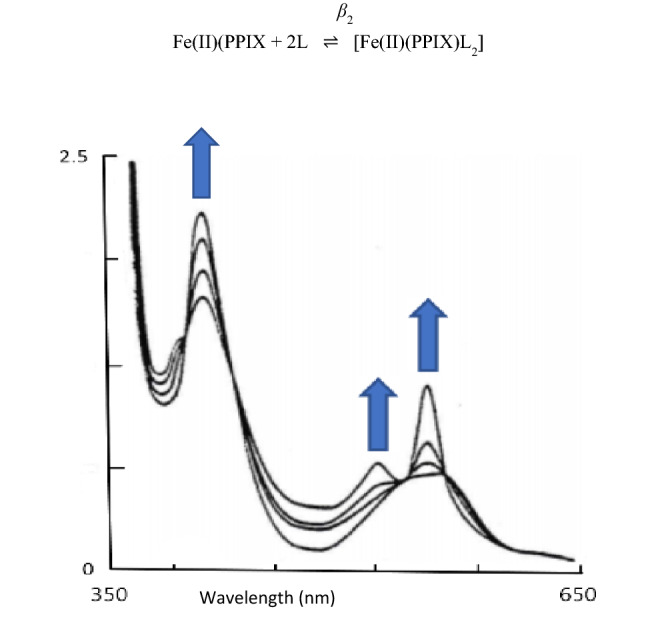

## Introduction

Iron(II) protoporphyrin IX, [Fe(PPIX)], is ubiquitous in natural enzymes [1–4]. Indeed [Fe(PPIX)] and related haems (iron porphyrin macrocycles) form the active centres in a wide range of biological molecules crucial for living organisms. These haem groups perform a diversity of roles such as oxygen transport (haemoglobin) and storage (myoglobin), electron transport (the cytochromes) and in the elimination of toxic and unwanted compounds (cytochrome P_450_) [1–4]. The chemical properties of the iron in the haem are modulated both by the porphyrin and by the nature of the axial ligands [4–6]. The manner in which the immediate environment of the metal is influenced by electron delocalization on the macrocycle and the nature of the axial ligation in iron porphyrin complexes has been much discussed for over 50 years [1–4, 7–14]. Many structures/molecules containing natural and synthetic haems have been studied to obtain clues to porphyrin metal bonding interactions and how axial ligands may control and/or modify this bonding [15–20].

We have carried out extensive studies on [Fe(PPIX)] chemistry using Mὃssbauer and optical spectroscopies [21–49]. In those studies we have demonstrated that by selecting the axial ligand, both the spin state of the [Fe(II)(PPIX)] and [Fe(III)(PPIX)] complexes and their geometry can be varied/controlled. In complementary studies we have applied the understanding gained; (1) to haem peptides derived from cytochrome c [50–52]; (2) to the role of [Fe(PPIX)] in porphyromonas gingivalis [47, 53–58] and other oral anaerobes [59, 60]; and (3) to haem-antimalarial complexes of pharmacological interest [61, 62].

Amongst the many studies on low-spin six-coordinate [Fe(II)(Por)L_2_] (where Por = porphyrin and where *L* the axial ligands are nitrogenous aromatic or aliphatic ligands), are a number that contain crystallographic and/or Mossbauer spectroscopic data. One such study that considered ligand orientation control gave amongst the major conclusions the fact that Mӧssbauer spectra provide a probe for ligand orientation when structural data may not be available [20]. The same paper presents a critical overview of the orientations of planar axial ligands in bis-ligated haems of both iron(II) and iron(III). This paper summarises and discusses a rich literature of crystal structures comparing the relative orientation of the two axial planar ligands to each other and, also to the four nitrogen atoms of the porphyrin core; in addition, it gives the Mӧssbauer parameters of the same complexes [20].

We have previously reported studies on a wide range of nitrogenous ligands binding to [Fe(II)(PPIX)] [43, 44]. These studies covered the binding of [Fe(II)(PPIX)] to pyridine, substituted pyridines, imidazoles, aliphatic amines and piperidine. The results were compared to previous literature on binding studies of pyridine and imidazoles to haems in non-aqueous systems and in so doing we summarised the many different factors that affect such binding. In our approach we used plots of pKa—log*β*_2_ and of Δ*E*_Q_ (Mössbauer parameter)—log*β*_2_ to analyse the results. This then innovative combination/display of the data was used to underpin our conclusions [43, 44]. It will be used here in a more complete way to take our previous results with those reported herein to allow an overview of the bonding properties of the axial ligands to [Fe(II)(PPIX)] and how they affect the properties of both the iron atom and the PPIX ligand. Herein we report the preparation of new low-spin six-coordinate [Fe(II)(PPIX)] complexes with a variety of aromatic nitrogenous ligands containing one, two or more N-atoms or another hetero atom in the aromatic ring to gain further insight to how variation in the bonding properties of such ligands can affect the [Fe(II)(PPIX)] entity.

This is of particular importance for cytochrome P_450_ mono-oxygenases which catalyse biological oxidations to hydroxylate a wide variety of compounds and act as an initial step in the detoxification of xenobiotics (such as drugs or hydrocarbons) in addition to the in vivo metabolism of compounds (for example steroids) [4, 5]. [Fe(PPIX)] is present as the catalytic centre in all cytochrome P_450_ mono-oxygenases; ligand binding studies to it allow the binding abilities of different nitrogenous ligands to be compared. Recently, a number of molecules containing azole, histidine, triazole or tetrazole ligands have been studied as inhibitors to cytochrome P_450_ in a variety of organisms [4, 5] and to understand the modus operandi of the binding of these inhibitors was a motivator in the choice of ligand studied in the current work and we will refer back to this in the discussion. In the cytochrome P_450_ mono-oxygenases the [Fe(PPIX)] is bound by an axial cysteine thiolate ligand and therefore only has one binding site available to bind to one inhibiting nitrogenous molecule. In our case our studies are on [Fe(II)(PPIX)] which can bind to two nitrogen ligands. However, the findings of this work are still relevant as they show the order of the binding abilities of the different nitrogenous ligands; this will be the same even in the presence of a different sixth ligand.

Herein stability constants were calculated from electronic absorption spectra and Mӧssbauer spectra were obtained from frozen solutions of the complexes. We also studied a small range of sterically hindered nitrogenous ligands to gain understanding on how steric effects may modify bonding. These investigations were directed at examining σ- and π-bonding effects as well as steric effects in the bonding of the axial ligands. Protoporphyrin IX was the porphyrin selected for the studies because it is the most widespread porphyrin found in natural proteins. Other reasons for its selection and its limitations have previously been discussed [39–41]. Following on from those studies our investigations were carried out at high pH where we have previously shown that a significant proportion of the [Fe(II)(PPIX)] is monomeric in the absence of nitrogenous bases. At lower pH values the aggregated [Fe(II)(PPIX)] are the dominant species in solution and such species complicate ligand binding studies [24, 32].

## Experimental

Haematin was purchased from Sigma and used without further purification. The nitrogen ligands (1–9, 13 and 14 presented in Table 1) were either purchased from Aldrich or supplied by ICI. The extremely sterically hindered ligands:- tert-butylamine (from Aldrich) was distilled from KOH prior to spectral use: 2-methylpyridine (from Aldrich) was fractionally distilled from KOH; tri-butylamine (from Aldrich) was distilled from KOH twice. The solvent diethyl glycerol was used without further purification.

**Table 1:** The electronic absorption sp ectra of the low-spin [Fe(PPIX)L_2_] complexes (where L = nitrogenous ligand) at pH 12, *λ*_max_ show Soret, *β* and *α* bands

Nitrogenous ligand and structure	Band Maxima Soret (nm)	Band Maxima *β* (nm)	Band Maxima α (nm)
1. 4(3) pyrimidone	418	523	554
2. 5-methyl pyrimidone	417	526	557
3. 2-methyl pyrazine-(5-methylpyrimidin-4(3H)-one)	415	527	558
4. 2-methoxypyrazine	419	530	564
5. 3-methylpyridazine	421	530	564
6. Thiazole	418	525	558
7. Oxazole	418	529	558
8. 4-n-butyl 1,2,4-triazole	417	525	556
9. 4-amino1,2,4-triazole	415	524	556
10. 2-methylimidazole^a^	418	525	554
11. Tertiarybutylamine^a^	416	521	552
12. 2-methylpyridine	418	528	560
13. 1-(2,4 dichlorophenyl)-2-(1,2,4-triazol-1-yl)-3-hydroxy-4,4-dimethylpentane	416	525	556
14. Methyl 2-(1,2,3,4-tetrazol-3-yl)-propionate	419	530	558

^a^Spectra taken in 50:50 aqueous:diethylglycerol at 77 K; at room temperature the spectra are those that of high-spin five-coordinate [Fe(PPIX)L] species. The spectra changes as the temperature decreases

Heamatin was first dissolved in NaOH (0.1 M) and then diluted to the desired concentration (~ 10^–5^ M) with NaOH to give a solution of final pH = 12. The heamatin was reduced to PPXIFe(II) with a slight excess of solid sodium dithionite.

Electronic absorption spectra were obtained using a DU-7 spectrophotometer (Beckman) between 350 and 750 nm.

Spectrophotometric titrations (at 293 K) were carried out anaerobically using serial addition of degassed solutions of the desired ligands. Small volumes (~ 20 μl) of a stock solution (either neat compound or suitably diluted solution) of ligand were serially added to ~ 3 ml of PPXIFe(II) solution, the precise volume being determined by the weight assuming a solution density of 1 g/cm^3^. Spectra were recorded three minutes after each addition to allow equilibrium to be established. The spectroscopic data were analysed by transforming the ligand binding curve utilising a Hill plot from which both the Hill coefficient and the log β_2_ values could be obtained. The values of these parameters quoted are the average of three experiments.

For the extremely sterically hindered ligands:- tert-butylamine, 2-methylpyridine and tri-butylamine the visible spectra at room temperature were measured according to the method outlined above. For the experiments carried out down to 77 K approximately 3 ml of reduced haematin and a 50% (V/V) aqueous glycerol solution (containing the sterically hindered ligand) was placed in a quartz cell and placed in a cryostat designed in our laboratories. The cryostat was then fitted into the spectrometer. The spectra were observed to change from those of typical high-spin [Fe(II)(PPIX)L] complexes to six-coordinate low-spin [Fe(II)(PPIX)L_2_] complexes as the temperature started to decrease. The spectrophotometric titrations for these ligands were carried out as above for the other ligands but with excess of ligand where necessary.

Mὃssbauer spectra were recorded on concentrated frozen solutions at 78 K. The Mὃssbauer spectrometer and experimental details have previously been described [43, 44].

## Results and discussion

In this work fifteen nitrogenous ligands were studied with [Fe(PPIX)]; the absorption peaks in the visible region of them are presented in Table 1.

### Visible spectra

From the known iron(II)porphyrin crystal structures [6–18], a useful approximation can be formulated, namely: “the porphyrin ring is essentially a plane and the iron atom in it is subjected to D_4h_ symmetry”. The iron porphyrin spectral bands that appear in the visible region are a consequence of extensive delocalization of π electrons on the porphyrin and we have previously discussed such spectra in detail [43, 44] and shown typical spectra [43]. The spectra recorded in Table 1 are similar in appearance to those and so additional figures are not necessary.

In Table 1 the spectra reported are similar to those we previously reported [43, 44] and to those reported by others [63] where we noted that the Soret band of porphyrin iron(II) complexes coordinated to aliphatic ligands shifts to longer wavelengths [44] while with unsaturated ligands (π-bonded systems) the Soret band moves towards shorter wavelengths [43]. So, as electron density is donated to the iron by/from the saturated ligands it is accumulated in the z direction and this will only have a slight effect on the spectrum (not affecting the π electrons of the porphyrin nitrogen atoms in the xy plane) [64]. In contrast when unsaturated ligands bind to the iron the metal *t*_2g_ orbitals (*d*_*yz*_, *d*_*xz*_) are involved in π-bonding to them modifying their electron density. This results in a decrease in the overlap of metal *t*_2g_ orbitals with the π orbitals of the porphyrin ring (via the porphyrin nitrogen atoms) leading to the shift of the Soret band to shorter wavelengths [43, 44, 63–67].

We demonstrated that some of the unsaturated ligand complexes manifested spectra in which the Soret band appears at longer wavelength (substituted imidazoles) [43]. For imidazole we suggested that the reason is that it has better σ-donating abilities than the other aromatic ligands. In addition, imidazole ligands can approach the iron closer than 6-membered rings as they experience less steric hindrance [44]. In this work 3-methylpyridazine also manifests a Soret band at longer wavelength (see Table 1), this is a weak ligand because of the presence of two adjacent nitrogen atoms on the ring, as one bonds the lone pair electrons on the other must be repelled by interactions between itself and the π-cloud of the porphyrin ring, and or by steric interactions involving the methyl group as well as the non-bonding N atom. This ligand is therefore forced away from the iron(II) centre and will behave as a weak ligand (vide infra stability constants in Table 2).

**Table 2:** Hill coefficient, ligand concentration at 50% saturation, stability constant of the low-spin [Fe(TPP)L_2_] complexes, and the p*K*_a_ of the free nitrogenous ligand involved

Nitrogenous Ligand	Hill coefficient *h*	50% saturation (M)	Stability constant (log *β*_2_ M^2^)	p*K*_a_
1. 4(3) pyrimidone	1.31	0.07	2.3	1.6
2. 5-methyl pyrimidone	1.72	4 × 10^−3^	4.8	1.9
3. 2-methyl pyrazine	1.90	1.3 × 10^−3^	5.7	1.45
4. 2-methoxypyrazine	2.3	7.5 × 10^−3^	6.2	0.75
5. 3-methylpyridazine	1.25	0.07	2.3	3.46
6. Thiazole	1.90	3.89 × 10^–3^	4.82	2.44
7. Oxazole	2.1	4.16 × 10^–3^	4.76	1.3
8. 4-*n*-butyl 1,2,4-triazole	1.15	0.08	2.2	2.3
9. 4-amino1,2,4-triazole	1.34	0.06	2.4	^b^
10. 2-methylimidazole^a^	1.9	0.043	2.7	
11. Tertiarybutylamine^a^	1.15	0.33	0.96	
12. 2-methylpyridine^c^				
13. 1-(2,4 dichlorophenyl)-2-(1,2,4-triazol-1-yl)-3-hydroxy-4,4-dimethylpentane	2.0	5 × 10^–3^	8.6	^b^
14. Methyl 2-(1,2,3,4-tetrazol-3-yl)-propionate	1.2	0.178	1.52	0.316

^a^The Hill coefficients of these ligand titrations are when excess ligand is added

^b^See ref [71] calculated pK_a_ for 1,2, 4-triazoles are 2.45

^c^The Hill coefficient and stability constant ect were not calculated for this ligand; see discussion in the visible spectra section

It is apparent from Table 1 that the heterocyclic ligands, that include in the carbon rings two different element types, oxygen plus nitrogen (in oxazole) and sulphur plus nitrogen (in thiazole), generate spectra very similar to those of the nitrogen bases. This provides evidence that the binding between [Fe(II)(PPIX)] and these ligands has taken place in a similar manner to those of the nitrogen bases.

Thus, the visible spectra of these [Fe(II)(PPIX)L_2_] compounds have been characterised and have provided insight into the electron density distribution between σ- and π-bonding in the z direction. Low-spin octahedral complexes are indicated in all cases.

Of the sterically hindered ligands only 2-methyl pyridine, manifested evidence of a low-spin complex from its visible spectrum at room temperature. This was a surprising finding as it was expected that the adjacent methyl group would cause steric hindrance to the binding and a high-spin five coordinate complex may have formed; as we could not rule out that there was an impurity present that generated the low-spin complex we did not derive binding constants for this complex. However, as will be discussed later we did observe a low-spin complex in its Mossbauer spectrum.

**Fig. 1:**
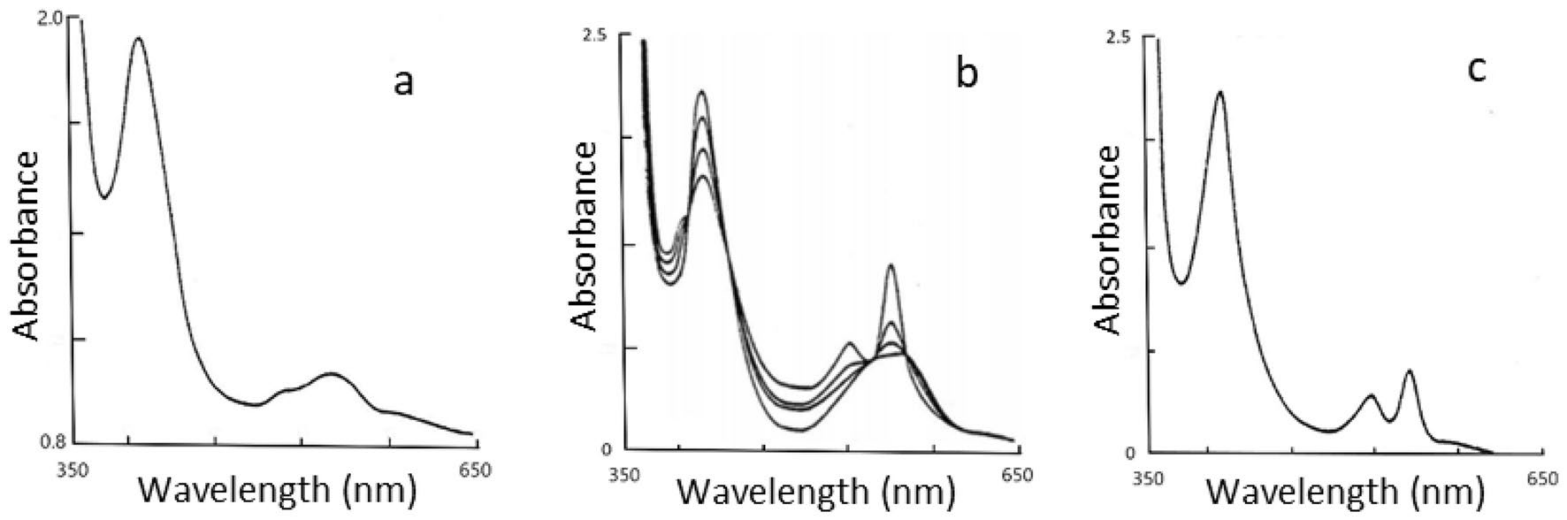
The electronic absorption spectra of [Fe(II)(PPIX)] in the presence of a large excess of 2-MeIm in 50:50v water/-glycerol. **A** Five coordinate [Fe(II)(PPIX)(2-MeIm)] at room temperature; **B** Changes in the electronic absorption spectra that occurred as the temperature was lowered to 78° K; **C** Six coordinate [Fe(II)(PPIX)(2-MeIm_2_)] at78° K

Two other ligands 2-methylimidazole and tertiary butylamine only gave the spectra of high-spin five-coordinate [Fe(PPIX)L] species at room temperature. However, by cooling the ligands with [Fe(PPIX)] in 50%(V/V) aqueous glycerol to 78 K the spectra of both were seen to change to low-spin complexes as recorded in Table 1 (see in Fig. 1). The last sterically hindered ligand we studied was tri-butylamine; we found no evidence for any kind of complex formation with this ligand, and its frozen solution Mossbauer spectrum manifested only the presence of [Fe(PPIX)]. Thus, the presence of three bulky butyl groups successfully repels the porphyrin plane and inhibit the amine nitrogen binding to the [Fe(PPIX)].

### Spectrophotometric ligand titrations

We have previously presented a typical spectrophotometric titration of the [Fe(II)PPIX] (reduced by dithionite in the presence of dithionite with imidazole [43]. Isosbestic points were seen at 402 nm, 462 and 568 nm respectively. Similar such spectral changes occur in all ligands studied in this work but are not illustrated herein; the reader is referred to reference [43] to see typical spectra of such titrations and the definition of *β*_2_. Though to help the reader, we also define *β*_2_ herein.

Falk et al*.* [44] studied the binding of some pyridine axial ligands to iron(II) haems in an attempt to distinguish between σ- and π-bonding effects. In their studies they observed no evidence for stepwise addition of the ligands thus

1 $${\text{Fe}}\left( {{\text{II}}} \right)\left( {{\text{PPIX}}} \right) \; + \;2L\overset {\beta_{{2}} } \rightleftharpoons \left[ {{\text{Fe}}\left( {{\text{II}}} \right)\left( {{\text{PPIX}}} \right)L_{2} } \right]$$

is the dominant reaction where *β*_2_ is defined from Eq. (2)

$${\text{Fe}}\left( {{\text{II}}} \right)\left( {{\text{PPIX}}} \right)\; + \;{\text{L}}\overset {K_{{1}} } \rightleftharpoons \left[ {{\text{Fe}}\left( {{\text{II}}} \right)\left( {{\text{PPIX}}} \right){\text{L}}} \right]$$

2 $$\left[ {{\text{Fe}}\left( {{\text{II}}} \right)\left( {{\text{PPIX}}} \right){\text{L}}} \right] + {\text{L}}\overset {K_{{2}} } \rightleftharpoons \left[ {{\text{Fe}}\left( {{\text{II}}} \right)\left( {{\text{PPIX}}} \right){\text{L}}_{{2}} } \right]$$

*K*_2_*» K*_1_; *β*_2_ = *K*_1_ *K*_2_.

**Fig. 2:**
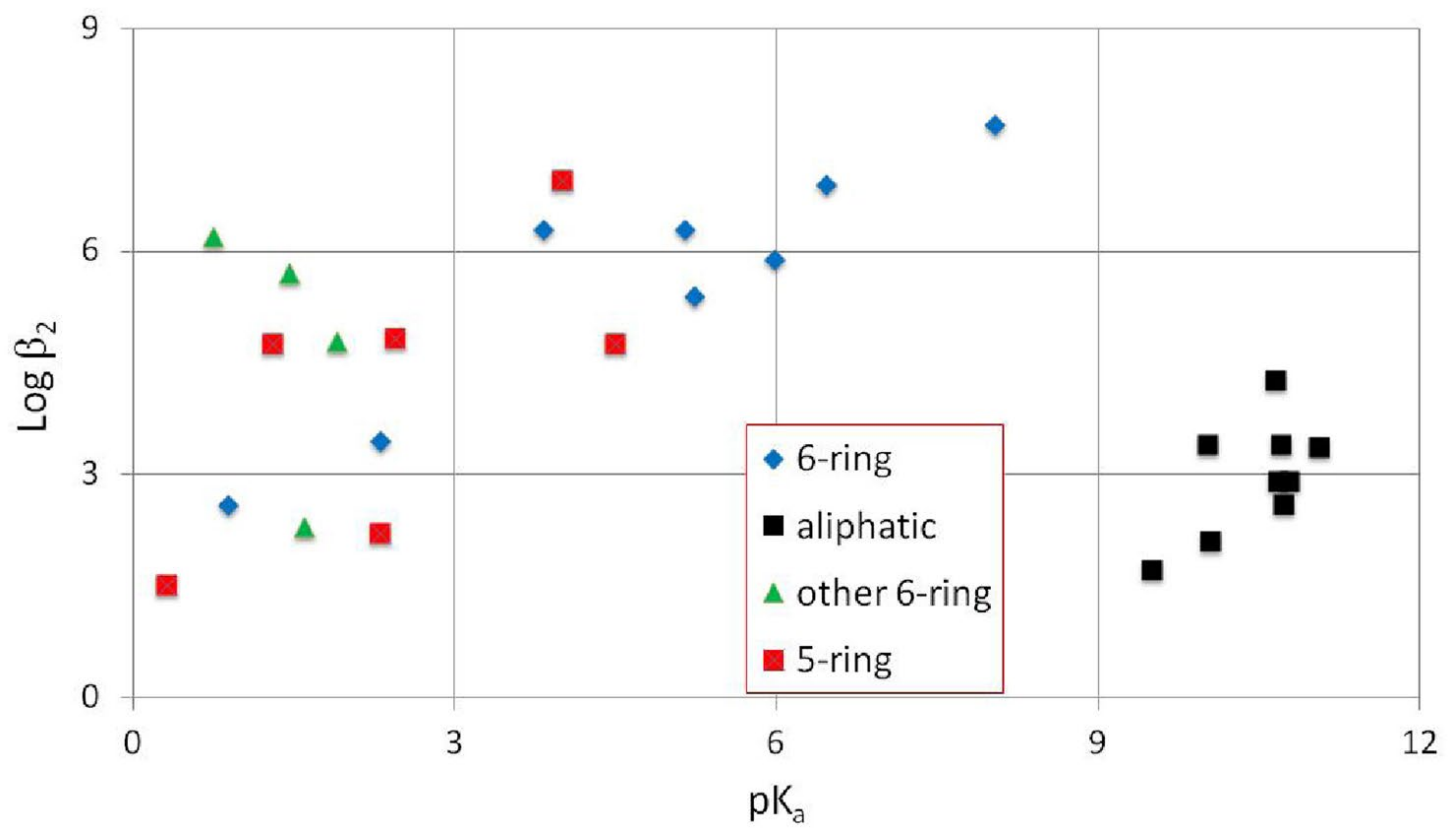
A plot of log *β*_2_ values against the p*K*_a_ values of the free ligands. The pyridines (6-ring coloured blue) can be fitted to a trend line, the imidazole and 5-membered rings (coloured red) would straddle this trend line, whereas the other six-membered rings (green triangles) are widely spread across the trend. The aliphatic ligands shown in black are well separated and in a tight group

The equilibrium constants for the [Fe(II)(PPIX)] moiety with these nitrogenous ligands have been calculated from the titration curves. The Hill coefficient (*h*) the ligand concentration at 50% saturation and p*K*_a_’s of the free ligands involved are given in Table 2, and the log *β*_2_ values are plotted against the p*K*_a_ values in Fig. 2.

The Hill coefficients (*h*) were found from Hill plots of the type illustrated in ref. [43] and took values between 1 and 2.5. Values of (*h*) close to unity indicate independent binding of ligands in a stepwise manner. Values of (*h*) greater than unity indicate cooperative binding of ligands such that for a system where two ligands may bind to a central iron atom a value of *(h*) of 2 indicates that ligands bind in such a fashion that throughout the titration no complex with a single ligand bond exists. The value of (*h*) cannot exceed the number of incoming ligand molecules. Therefore, values of (*h*) greater than 2 indicate cooperative binding of ligands not only to the two coordination positions of the iron but also to either the porphyrin ring [68, 69] or through hydrogen bonding to already bound ligands (i.e. ligand self-association) [70]. Other factors affecting Hill coefficients have been previously discussed [43, 44].

In Fig. 2 the compounds studied are grouped in structural classes, e.g. the pyridines [43], aliphatic amines [44], the five-membered rings reported in this work (these would straddle a trend line of the pyridines and their trend line would be very similar to that of the pyridine ligands) and finally the four other six-membered rings which are at the left hand end of the pyridine trend line are more spread out, reflecting their more varied structures. It is apparent that the aliphatic ligands are well separated from the unsaturated five and six-membered ring ligands. The former ligands are pure σ-electron donating axial ligands and their binding constants to [Fe(PPIX)] are in the main lower than those of the unsaturated five and six-membered ring ligands (that are able to bond through a combination of both σ-electron donation and π-bonding). Where steric constraints do not greatly hinder binding (e.g. the aliphatic amines, the pyridines and the imidazoles) we note the trend within each group is that the higher the p*K*_a_ of the amine, the higher is the binding constant. In other words, increasing the affinity of a compound for protons increases its affinity for iron presumably because each bears a positive charge.

The exception to this trend is the group containing other six-membered aromatic rings. Although, this is a heterogeneous group of compounds, general features regulating the p*K*_a_ and the binding constants may be discerned. Two pointers in particular are noteworthy, i.e. whether the nitrogen atoms are ortho, meta or para to each other and whether the ligand is sterically hindered by the presence of a second nitrogen (ortho to it, e.g. 3-methyl pyridazine) that repels the porphyrin ring causing a lower binding constant.

Ligands 8, 9 and 13 in Table 1 are all substituted 1,2,4-triazoles. Although we do not have measured p*K*_a_ values for these ligands, there is a calculation in the literature suggesting they should have values of around 2.45 [71]. We did not include these three on the plot in Fig. 2 as we could not be sure about their exact p*K*_a_ values, however, compounds 8 and 9 would fit the trend line for the 5-membered rings quite well with p*K*_a_ values around 2.45 whereas compound 13 which has the highest binding constant of all the ligand studied would not. From this we believe that the binding constant for compound 13 is not just due to the presence of its five-membered ring, but also to other parts of its structure and we will return to this ligand later.

The 4(3) pyrimidone and pyridine-*N*-oxide [43] have similar values for log*β*_2._ The latter ligand must bind through its oxygen atom^25^ and from an examination of the structure of 4(3) pyrimidone it is apparent that the enol form of this molecule at pH 12 would bear a deprotonated oxygen through which presumably it binds to iron. The nitrogen, para to oxygen binds much less strongly than the nitrogen of pyridine as evidenced by the much lower p*K*_a_ (1.6) (as opposed to the p*K*_a_ 5.23 for pyridine). If binding is through a negatively charged oxygen, then it is reasonable that the Hill coefficient is lower than that for the neutral nitrogen ligands possibly indicating a lower stoichiometry at room temperature (compare Mὃssbauer spectroscopic data at 78° K, see discussion in the Mossbauer section below) [43].

In this context the spectral features of the [Fe(II)(PPIX)] compound with pyridine-*N*-oxide are in keeping with a high-spin environment (at room temperature) rather than the low-spin species seen with nitrogen ligands. When the ring bears a sterically unhindered N atom, an electron donating group and is neutral, then the binding affinity to the iron(II) is high as is the Hill coefficient (compounds 2–4 in Table 2).

A striking feature of all the nitrogen ligands (except 4(3) pyrimidine and pyridine-*N*-oxide [43], which bond through oxygen) irrespective of whether they are aliphatic or aromatic or indeed whatever the value of log *β*_2_ is that the Hill coefficient is close to two. This implies that during a titration the single-liganded complex is poorly populated. In other words, the binding of the second ligand is enhanced by the binding of the first. The reasons for this behaviour are not obvious; we have nevertheless offered a tentative explanation [43].

A paper that considered the binding of imidazole ligands to metalloporphyrins [72] is also relevant to this as it contains the somewhat surprising finding that the metal pπ-orbitals bind to the imidazole nitrogen pπ-orbitals. This conclusion which explained the stereochemistry of the known crystal structures would also be in keeping with the elevated values of log *β*_2_ found for the aromatic compounds in this work (see Table 1), compared with the aliphatic nitrogen ligands [44]. The finding of cooperative nitrogen binding reported here may have implications in the biological role of nitrogen ligands in haem proteins. A nitrogen base is almost invariably one of the axial ligands and in many cases, (for example in the electron transfer proteins) two axial nitrogen ligands are present.

Quinn et al. [73] found that there was a linear relationship between the equilibrium constant and p*K*_a_ for the hydrogen-bonded imidazoles (ImH) and that such relationships are absent for non- hydrogen-bonded imidazole derivatives. Their p*K*_a_’s have been determined in aqueous solution, where ImH…OH_2_ rather than ImH…ImH is the predominant mode of hydrogen bonding. They found that their value of K_eq_ as determined in non-hydrogen bonding solvents was apparently influenced more by the nature of the hydrogen bonding to coordinated imidazole than by the nature of any substituted substituent group on the ligand. This is an important point as in our work (and in ref. [63]) all the measurements were made at pH 12, where the 2-methylimidazole would bind not as 2-MeHIm, but rather as 2-MeIm either completely deprotonated or partially deprotonated with a solvated proton close by.

The stability constants and Hill coefficients are reported (in Table 2) for the complexes [Fe(PPIX)(2-MeHIm)_2_] and [Fe(PPIX)(^t^ButNH_2_)_2_] and they were also investigated for the analogous [Fe(TMPPS_4_)(L_2_] complexes (the latter are not reported any further here).

The 50% saturation of [Fe(PPIX)(2-MeHIm)] is higher than that for[Fe(PPIX)(^t^ButNH_2_)]. Similar results were obtained for [Fe(TMPPS_4_)] with these ligands. This might be explained in terms of the presence of electron π-back donation in the case of 2-methylimidazole and the absence of this phenomena in the case of the tert-butylamine (the latter ligand is an aliphatic amine).

It is noted that the addition of a large excess of sterically hindered ligands greater than 1 M to [Fe(PPIX)] results in a coordination change from penta-coordinate to hexacoordinate due to stepwise binding at the second axial ligand site. An increase in affinity for the second ligand has been attributed to reflect an exothermic enthalpy change of the equilibria [9]. The close spectral similarities between the hindered ligand at 78 K with unhindered ligands at room temperature provide support for a change in coordination of the hindered ligand complexes at low temperature.

Brault and Rougee [9, 10], have reported that weak ligands such as alcohol or water molecules can act as a second axial ligand to the iron porphyrin system. This is a fact that must be kept in mind when studying spectra taken from frozen solutions. To this end we can compare spectra to our previous studies where no possible ligands were present other than those of the aqueous solution itself [24, 32]. In this case reference spectra can be taken from our previous work on frozen solutions of [Fe(PPIX)] as a function of pH [24, 32]. Thus, in the case where ligands that are sterically hindered and weaker binding have been added, then by comparing their spectra we can tell if new compounds have formed.

Coleman. et. al. [12] and Brault et. al. [13] found that only one molecule of 2-methylimidazole was bound to ferrous haem in organic solvent. Wagner et. al. [14] observed low affinity for the binding of one equivalent of 2-methylimidazole and tert-butylamine in aqueous alcoholic solvents.

The observed differences in the equilibrium of iron(II) haems with hindered and unhindered ligands can be interpreted using steric arguments. The dominating factor has been shown to be the presence of bulky methyl groups situated on the α-carbon leading to the coordination of only one ligand to iron(II) haem at room temperature resulting in a high-spin iron(II)porphyrin complex [14]. Thus, steric repulsion of these methyl groups with the porphyrin planes is the driving force responsible for limiting coordination of a second axial ligand except in the case where the concentration of the ligands is increased.

In agreement with this hypothesis the spectroscopic titration of the high-spin electronic sites of the [Fe(PPIX)(2-MeIm)] and [Fe(PPIX)(^t^ButNH_2)_] at ambient temperature demonstrate their close spectral similarity to known high-spin ferrous haem proteins; these facts are relevant to the chemistries of the deoxymyoglobin and deoxyhemoglobin as we have previously discussed [38].

### Mὃssbauer spectroscopy

To aid in the understanding of the electronic environments around the iron(II) centres and how these are affected by the binding of ligands, Mὃssbauer spectra were collected on frozen solutions at 78° K. The spectra all consisted of sharp doublets and the parameters are presented in Table 3. The range found for the isomer shifts for the purely σ-bonding ligands is 0.41–0.52 mms^−1^ (all referenced to natural iron foil), and that for quadruple splittings (Δ*E*_Q_) was 1.03–1.40 mms^−1^ [44], whereas the isomer shift range for the unsaturated ligands reported previously [43] and in this work was 0.41–0.54 mms^−1^ and the quadruple splitting range was 0.91–1.20 mms^−1^. These values agree well with previously reported data [74].

**Table 3:** A ^57^Fe Mossbauer spectral parameters for low-spin [Fe(TPP)L_2_] complexes in frozen solution at 78° K

Nitrogenous ligand	*δ* (mm s^−1^)^a^	Δ*E*_Q_ (mm s^−1^)	Г (mm s^−1^)^b^
1. 4(3) pyrimidone	0.42 (1)	1.20 (1)	0.17 (1)
2. 5-methyl pyrimidone	0.42 (1)	0.98 (1)	0.14 (1)
3. 2-methyl pyrazine	0.41 (1)	1.07 (1)	0.18 (1)
4. 2-methoxypyrazine	0.48 (1)	1.14 (1)	0.13 (1)
5. 3-methylpyridazine	0.47 (1)	1.09 (1)	0.18 (1)
6. Thiazole	0.42 (1)	1.08 (1)	0.14 (2)
7. Oxazole	0.46 (1)	0.94 (1)	0.27 (1)
8. 4-n-butyl 1,2,4-triazole	0.45 (2)	1.00 (2)	0.14 (3)
9. 4-amino1,2,4-triazole	0.46 (1)	1.01 (1)	0.21 (1)
10. 2-methylimidazole^c^	0.51 (2)	0.96 (3)	0.16 (2)
11. Tertiarybutylamine^d^	0.54 (1)	1.09 (2)	0.17 (1)
12. 2-methylpyridine^d^	0.44 (1)	1.12 (1)	0.17 (1)
13. 1-(2,4 dichlorophenyl)-2-(1,2,4-triazol-1-yl)-3-hydroxy-4,4-dimethylpentane	0.45 (1)	0.91 (1)	0.30 (1)
14. Methyl 2-(1,2,3,4-tetrazol-3-yl)-propionate	0.46 (1)	1.07 (1)	0.14 (1)

^a^δ is relative to iron foil;

^b^Half width at half height

^c^This spectrum gave evidence for 17(3)% of this low-spin complex and 83IX(3)% of a five-coordinate high-spin complex believed to be [Fe(PPIX)(2-MeIm)]

^d^This spectrum gave evidence for 18(1)% of this low-spin complex and 53(1)% of a five-coordinate high-spin complex believed to be [Fe(PPIX)(^t^ButNH_2_)] and 24(1)% of the unreacted(none ligated to ^t^ButNH_2_) starting [Fe(PPIX)]

**Fig. 3:**
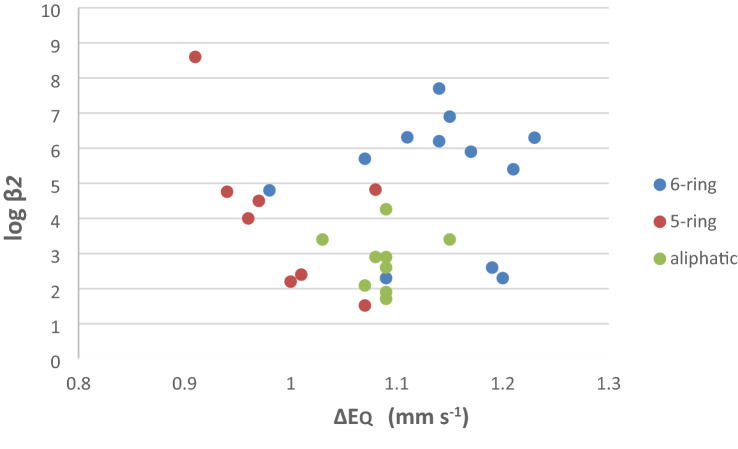
A plot of log *β*_2_ against quadrupole splitting (Δ*E*_Q_) for the Fe(II)(PPIX)L_2_ complexes. The six-membered rings mainly have the largest Δ*E*_Q_ values. The pyridine rings are high and to the right. The five-membered rings are predominantly on the left they have smaller Δ*E*_Q_ values and their shape and bonding properties allows them to get close to the Fe(II) atom. The aliphatic ligands are all at the bottom

It can be observed both in this work and previous reports [43, 44, 74] that there is little difference in the isomer shifts of these complexes, all are close to each other with a slight trend to smaller values for the imidazole rings and larger for piperidine. However, significant changes are observed in the quadrupole splitting (Δ*E*_Q_). For example, for the five-membered unsaturated ring oxazole a small Δ*E*_Q_ is observed, whereas for the six-membered ring piperidine a larger Δ*E*_Q_ is observed; the six-membered unsaturated rings lie in between these extremes. A plot of the log *β*_2_ values against the Δ*E*_Q_ values of the complexes is presented in Fig. 3.

We have previously discussed factors affecting low-spin [Fe(II)(PPIX)L_2_] Mὃssbauer parameters [43, 44]. As stated when the aliphatic ligands bind to low-spin [Fe(II)(PPIX)] moieties the resulting complexes manifest Δ*E*_Q_ values in the range 1.03–1.15 mms^−1^ (except for piperidine, 1.4 mms^−1^) and bind with iron via an sp^3^ hybrid orbital in a σ-bond [44]. The fact that this Δ*E*_Q_ range is small and the absolute magnitude is less than that for cyclic aliphatic compounds can be interpreted to indicate that these ligands show no evidence of steric repulsion with [Fe(II)(PPIX)]. For completeness, the Δ*E*_Q_ values of these ligands are plotted in Fig. 3 against their stability constants. The aliphatic ligands (indicated in green) are all at the bottom in Fig. 3. For the [Fe(II)(PPIX)L_2_] complexes where L = pyridine type ligands (indicated in blue in Fig. 3) [43], the Δ*E*_Q_ values were seen to be ordered and increasing with decreasing p*K*_a_ values, except for isoquinoline, which was out of its expected position (see Table 4 in ref. [43]). It appears that isoquinoline has a much greater π-acceptor capacity than pyridine; this has been discussed by others [73] and is due to the greater delocalization of its π-electrons. The [Fe(II)(PPIX)L_2_] complexes where L = pyridine type ligands all fall towards the upper right of Fig. 3. Another observation that stands out in Fig. 3 is that for the 6-ring ligands (all indicated in blue in Fig. 3) there are two clusters of points. The first cluster referring to 3 compounds has rather low log *β*_2_ values (between 2.2 and 2.50) whereas the other cluster has much higher log *β*_2_. The same can also be recognized for the 5-ring compounds. The 6-ring compounds with low log *β*_2_ values are 3-methyl pyridazine and (3)-pyrimidone and pyridine-*N*-oxide (see Table 2 in ref. [43]). The cluster (3 points) of 5-ring compounds (indicated in red in Fig. 3) with low log *β*_2_ values are triazole, thiazole and tetrazole ligands.

Figure 3 shows the relationship between log *β*_2_ and ΔE_Q_. The ΔE_Q_ in this context gives an estimate of the electron imbalance between the four nitrogen atoms of the porphyrin on the one hand and the two nitrogen atoms of the axial ligands on the other. The major factor which affects Δ*E*_Q_ is the distance of the axial nitrogen ligands to the iron, this in turn will reflect the orbital composition of the bond (and bond strength) and any steric interactions. From the position of the compounds in Fig. 3 it is apparent that they can be grouped into four groups or classes. These are:

1. The aliphatic ones (shown as green circles in Fig. 3) which manifest little or no steric hindrance have Δ*E*_Q_ around 1.1 mm s^−1^. These compounds bind to iron solely via σ-bonding and their binding constants are low. Piperidine is also a pure σ-bonding ligand but because of steric hindrance is on the extreme right of the group as it manifests the greatest quadrupole splitting.
2. The five-membered ring ligands (shown as res circles in Fig. 3) manifest the smallest orbital imbalance. Therefore, they can (a) get close to the iron, and (b) accept some iron 3d electron density into their π^*^-orbitals. As a result, some of them have higher binding constants than the aliphatic ligands. These five-membered rings all lie towards the left of Fig. 3 except for thiazole which contains a S atom in the ring.
3. The six-membered ring ligands experience greater steric hindrance than the five-membered ring ligands and although they are better π-acceptors (which elevates their binding constants), they cannot approach the iron so closely. Thus, they have greater electron imbalance than the five-membered ring ligands and hence exhibit greater Δ*E*_Q_ values. These six-membered rings can be further split into two groups by their position in Fig. 3. The pyridine type ligands and the two pyrazine ligands form a group at the upper right of the figure, the second group of three compounds (compounds 1 and 5 of Table 3 and pyridine *n* oxide) have log β_2_ values around 2 compound 2 of table 5 has a larger log β_2_ value but it has the smallest Δ*E*_Q_ value than any of the other six-membered rings.

In a follow up work we will attempt to quantify/understand the control of the bonding in terms of σ- and π-bonding on both the axial ligands and the porphyrin nitrogen atoms. However, it is useful to consider the ligands presented herein (in Table 1) in greater detail at this point.

### The ligands presented in Table 1

Compound 1 is the ligand 4(3H) pyrimidone, this is a weak σ-donor with a p*K*_a_ of 1.6, while the Mὃssbauer spectrum of its [Fe(II)(PPIX)L_2_] complex indicates it is hexacoordinate at 78° *K* with a Δ*E*_*Q*_ of 1.20 mm s^−1^. Here the presence of the oxygen on the ring causes an electron withdrawing (inductive) effect, this makes this ligand a poor σ-donor, but a strong π-acceptor taking electrons from either the *3d*_*xz*_ or *3d*_*yz*_ orbitals of the metal. (The nature of the sixth ligand is again open to question just as in the case for pyridine *n* oxide [63]). This complex appears towards the bottom of Fig. 3 well below the pyridine complexes.

Compound 2 is 5-methyl pyrimidine is another weak σ-donor; this has a p*K*_a_ of 1.9 and its [Fe(II)PPIX] complex has a Δ*E*_Q_ of 0.98 mm s^−1^. From a consideration of the structure of this ligand in comparison to that of pyridine, it is a better π-acceptor, but poorer σ-donor, and is no more sterically hindered when it binds to [Fe(II)PPIX]. This allows the ligand to approach the haem easily and it manifests a high stability constant. The fact that it has a similar Δ*E*_Q_ value to pyridine [43] must reflect the presence of a 5-methyl group on the second nitrogen in the ring. The presence of an alkyl group in the 3 or 5 position will inhibit the metal–ligand π^*^ delocalization. The electrons in the *d*_*xz*_ and *d*_*yz*_ orbitals will then cause a negative contribution to *V*_*z*z_, **(**where *V*_zz_ is the principal direction of the electronic field gradient; we have discussed this and the effect on it at the iron atom in the porphyrin by a variety of axially binding nitrogenous ligands [43, 44]) and this is concomitant with the small observed Δ*E*_Q_ value for this complex. This complex is found to the left of the pyridine complexes and a little below them in Fig. 3.

Compounds 3 and 4 form [Fe(II)(PPIX)L_2_] complexes with L = pyrazine ligands these are 2-methyl pyrazine with a p*K*_a_ of 1.45 and 2-methoxy pyrazine (p*K*_a_ 0.75). The presence of a methyl group on the ring (electron donating) makes the former ligand a poor π-acceptor [75] whereas the presence of the methoxy group (electron withdrawing) on the ring results in the opposite effect. The latter also affects the spreading of electron density into the π^*^ ring orbitals from the haem and this increases Δ*E*_Q_ to a value larger than that caused by 2-methyl pyrazine. These two [Fe(II)(PPIX)L_2_] complexes sit close to the pyridine complexes in Fig. 3.

Compound 5 is 3-methyl pyridazine (p*K*_a_ of 3.46), has a Δ*E*_Q_ value of 1.09 mms^−1^. With this p*K*_a_ one might expect this ligand to have similar properties to 4-chloropyridine as it has a similar p*K*_a_ (compare 3.83). However, the presence of the adjacent nitrogen atom causes a steric repulsion to the π-cloud of the porphyrin. This manifests in the lower stability constant. At low temperature 78° K when the ligand and is bound to [Fe(II)(PPIX)] a small Δ*E*_Q_ (less than that of 4-chloropyridine) is observed; this can be explained in the same manner as for 5-methyl pyrimidine (vide infra). This [Fe(II)(PPIX)L_2_] complex is the furthest from the pyridine complexes in Fig. 3 situated towards the lower left.

Compounds 6 and 7 are five-membered heterocyclic ligands, thiazole (pk_a_ 2.44) and oxazole (p*K*_a_ 1.3), they have Δ*E*_Q_ values of 1.08 and 0.94 mms^−1^ respectively. Oxazole binds to the haem via the nitrogen atom and is able to accept more π-electron density than the thiazole, as the sulphur atom in the latter ring will be comparatively electron rich [76]. Thus, it can be argued that the oxazole is the better π-acceptor and this agrees with the p*K*_a_ values observed. These ligands can in addition (compare the stability constants) approach close to [Fe(II)PPIX] as they are similar in size to imidazole [43]. The [Fe(II)(PPIX)L_2_] oxazole complex manifests the second smallest Δ*E*_Q_ value of all the nitrogenous ligands shown in Fig. 3, it is situated to the centre left. The thiazole complex is at the same level but is the furthest to the right of the five-membered rings.

Compounds 8 and 9 are respectively 4-*n*-butyl-1,2,4-triazole and 4-amino-1,2,4-triazole both contain nitrogen atoms that are adjacent to nitrogen atoms that will compete for the site of ligation when they form [Fe(II)(PPIX)L_2_] compounds. Also, the lone pairs of electrons on the adjacent nitrogen will sterically oppose ligation as discussed for 3-methyl pyridazine. This is manifest in their weak binding constants to [Fe(II)(PPIX)]. These five-membered rings once bound to [Fe(II)(PPIX)] generate small Δ*E*_Q_ values similar to that of imidazole [63] with the 3rd nitrogen in the ring having little effect. The amino group of the 4-amino-1,2,4-triazole acting as a ligand and can be ruled out by consideration of the visible spectrum the Soret band is positioned at 415 nm, typical of an aromatic nitrogen [77]. These two [Fe(II)(PPIX)L_2_] compounds appear close together on the bottom left of Fig. 3.

Compound 13 is the 1-(2,4 dichlorophenyl)-2-(1,2,4-triazol-1-yl-3-hydroxy-4,4-dimethylpentane ligand. The resulting [Fe(II)(PPIX)L_2_] complex of this ligand is a very interesting compound as it has the highest stability constant of all the ligands studied in this work and has the smallest Δ*E*_Q_ value. As discussed earlier herein it has a calculated p*K*_a_ value [71] around the same as the other two -1,2,4-triazole complexes discussed in the previous paragraph, and as its stability constant is so high it cannot be due to the p*K*_a_ value alone and must be due in part to another property of the ligand. From considering the structure of this complex it is clear, that it is the nitrogen in the four position on the 1,2,4-triazoyl ring that binds to the iron atom in the haem. This is the only ligand used in this study which possesses a substituent group containing an aromatic ring capable of interacting (stacking) with the porphyrin ring. We suggest therefore that the high stability constant derives in part from the stabilisation of the complex through such interactions. Such interactions have been observed elsewhere for instance in the structure of [Fe(TPP)(OCl_3_)] which contains a m-xylene solvate molecule interacting with the porphyrin [68]. This ligand substitution pattern is such that the other free nitrogen does not interfere in the bonding unlike the other triazoles. Moreover, the smaller Δ*E*_Q_ value than that of imidazole suggests that it is a better σ-donor, however, it may be due to the stacking interaction of the aromatic ring forcing the lone pair in the nitrogen closer to the Fe atom. This [Fe(II)(PPIX)L_2_] compound appears close to the extreme top left of Fig. 3.

Compound 14 the tetrazole ligand (also an aromatic ring that is considered to be a good π-acceptor) appears at the bottom of all the five-membered rings also on the left in Fig. 3. The presence of the bulky group on the ring causes this ligand to be sterically hindered when it approaches the haem. From the structure of the tetrazole it can be seen that this ligand has only the nitrogen atom (N_4_) free of the steric repulsion caused by the bulky side chain, and thus available for bonding to [Fe(II)(PPIX)]. Even this nitrogen atom will be sterically hindered in its approach to the haem from the lone pair of the N atom in the 3 position on the ring. The small stability constant observed is in keeping with these arguments.

Mӧssbauer spectroscopy is a powerful tool to complement the visible spectroscopic studies on the sterically hindered ligands binding to [Fe(PPIX)]. Such measurements provide diagnostic data (particularly in the light of our previous studies on frozen solution that can be used as reference spectra) [23, 24, 26, 27, 32, 33, 36, 38, 41–44], on both the iron oxidation state and spin state; they can give significant information on the number of coordinated axial ligands [12]. The Mӧssbauer spectra of the three sterically hindered nitrogenous ligands all give evidence for low-spin [Fe(PPIX)L_2_] complexes. For the 2-methylpyridine ligand (compound 10) the resulting [Fe(II)(PPIX)L_2_] complex is in agreement with the room temperature absorption spectrum, moreover as no trace of any other species was present in the Mӧssbauer spectrum, we do not believe that this spectrum was generated by an impurity. Structural evidence backing this finding will be presented later in this work. The other two sterically hindered nitrogenous ligands compounds 11 and 12 the 2-methyl imidazole and tertiary butylamine ligands both have strong evidence of low-spin [Fe(PPIX)(L_2_)] complexes albeit with proportions of unreacted starting material and a five-coordinate high-spin [Fe(PPIX)L] species. In both cases this is supportive data to the low temperature electronic absorption data where a change to a low-spin complex was observed (see the electronic absorption spectra in Fig. 1). Interestingly in all three cases the [Fe(PPIX)(L_2_)] complexes of the sterically hindered ligands all have Δ*E*_Q_ values that are very close to similar non-sterically hindered ligands, though their chemical shift values are a little higher [43, 44].

A number of crystal structures have been reported for six coordinate low-spin iron(II) porphyrin complexes. Typical Fe–N (where N is an axial nitrogen ligand,) bond lengths depend on the nature of the nitrogen ligand. For aromatic ligands such as pyridine Fe–N = 2.10(1) Å in [Fe(TPP)(py)(CO)] [78], 2.037(1) Å and 2.039(1) Å in [Fe(TPP)(py)_2_] [79, 80]. In a substituted TPP porphyrin the (5,15-[2,2′-(dodecanediamido) diphenyl]: a,cx-l0,20-bis(o-pivaloylaminophenyl)porphyrin = Por) complex [Fe(Por)(CO)(1-MeIm)] [81] the Fe-N_Im_ distance is 2.062(5) Å is long, whereas for 1-MeIm in [Fe(TPP)(1-MeIm)_2_] [82] Fe-N_Im =_ 2.014(5) Å. Shorter axial bonds are also apparent in [Fe(TPP)(1-VinylIm)_2_] [83] Fe-N_Im_ = 2.004(2) Å and in [Fe(TPP)(1-BzLIm)_2_] [83] Fe–N = 2.017(4). Even shorter Fe-N_Im_ axial bonds are found in [Fe(TpivPp)(1-MeIm)_2_] [84] Fe-N_Im_ = 1.9958(19) Å and 1.9921(18) Å and in [Fe(TpivPp)(1-VinylIm)_2_] [84] the Fe-N_Im_ distances are 1.9979(19) Å and 1.9866(19) Å. There is only one example of a saturated axial ligand piperidine. For piperidine the analogous distance is [Fe(TPP)(pip)_2_] [85] is 2.127(3) Å. The bond lengths to iron in [Fe(TPP)(L_2_)] (L = nitrogen ligand) order as follows: 1R-Im < pyridine < piperidine. This is as expected as imidazole is the best σ-donor of the first two while piperidine, which is only able to σ-donate, is sterically hindered. It is therefore apparent to this point that the known crystal structures are in agreement with the bonding implications discussed herein. These small distances support our earlier findings [43, 44] and those of this work, and it follows on from this that such distances are similar to those found in naturally occurring haem proteins (which contain histidine) and that this is the reason for the widespread use of histidine as axial ligands to iron porphyrins in haem proteins. [2, 41]. In further support we have reported the Mӧssbauer parameters for the [Fe(II)(PPIX)L_2_] complexes where L = histidine and histidine type ligands (Δ*E*_Q_ values are in the range 0.88 mm s^−1^ to 1.04 mm s^−1^) [41] and where L = imidazole ligands (Δ*E*_Q_ values are in the range 0.95–1.04 mm s^−1^) [38] in a variety of different solvents. These values compare well with the reported Δ*E*_Q_ value of 1.04(3) mm s^−1^ for reduced cytochrome *b*_5_ [86].

It is also important to bear in mind when recording Mӧssbauer parameters from six-coordinate low-spin iron(II) porphyrin complexes (and also six-coordinate low-spin iron(III) porphyrin complexes) in frozen solutions that the nature of the solution and the nature of the substituent groups on the axial ligands can affect the bonding of the axial ligands to the porphyrin [34, 35, 37–41]. This can result for instance in the Δ*E*_Q_ values of the complex varying with the pH of the solution before it was frozen or varying with the solution itself (whether it was aqueous or non-aqueous). Such different solutions can affect the hydrogen bonding to and around the axial ligand. For ligands such as histidine and imidazole changes in the hydrogen bonding can lead to changes in the orientation of the planes of the two axial ligands to each other and also to their orientation with the porphyrin nitrogen to iron bonds [39, 40]. We have discussed such effects in detail previously [34–41]. It is obvious from this discussion that any changes in the solvent can also affect the orientation of both conjugated and non-conjugated substituent groups on both aliphatic and aromatic nitrogenous ligands.

### Relevance to cytochrome P_450_ mono-oxygenases inhibitors

In the last twenty-five years there has been a great deal of interest in designing molecules to block/inhibit the function of cytochrome P_450_ mono-oxygenases as a possible way of treating a variety of cancers and certain diseases such as tuberculosis. In particular, the enzymes involved in the biosynthesis of steroid hormones have become targets for therapeutic intervention [87]. Many of the nitrogenous ligands studied in this paper are relevant to these studies as they form the binding groups of the inhibitors to the [Fe(PPIX)] moiety found in the P_450_ mono-oxygenases. Although it is beyond the scope of our work to discuss them all we will show how studies of the kind reported herein can aid the understanding of both in viva and in vitro therapeutic results.

One such enzyme is aromatase (P_450Arom_)_,_ an enzyme complex formed from cytochrome P_450_ haemoprotein and an NADPH cytochrome P_450_ reductase which catalyses the final step in the steroidogenic pathway for the synthesis of oestrogens from cholesterol [87]. Aromatase inhibitors were shown to be useful in the second line therapy of oestrogen-dependent breast cancer in post-menopausal women [88]. Non-steroidal P_450Arom_ inhibitors include liarozole an azoyl-substituted benzimidazole [89] and the 1-[(benzofuran-2-yl)phenylmethyl]-imidazoles [90], which are active at IC_50_ < 10 nM. The synthesis of a series of 1-[(benzofuran-2-yl)phenylmethyl]-triazoles and tetrazoles was described and the compounds were tested for human placental aromatase inhibition in vitro to compare with the known imidazoles [87]. The triazoles were shown to be better inhibitors than the tetrazoles but both series were well down on the imidazole compounds by tenfold or 100-fold respectively. The reason for the decrease in performance of the triazoles relative to the imidazoles was suggested to be due to the additional nitrogen atom in the heterocyclic ring reducing the coordination potential of the nitrogen binding to the Fe atom as a result of the electron withdrawing effect of additional nitrogen atom [87]. This effect was said to be enhanced by the additional N atom in the tetrazole ring as there are then two additional nitrogen’s withdrawing electron density from both sides of the coordinating N atom [87]. However, these authors note that there are several other potent inhibitors of aromatase that contain a triazole moiety [87]. The binding constant results of our work described herein on the [Fe(II)(PPIX)L_2_] complexes for two of the triazoles (2.0–2.4), with a value of 1.52 for the tetrazole and our previous work on imidazoles (4.0) are in good agreement with the results of Vinh et al. [87], though the result for the triazole complex 12 of 8.6 shows a much higher binding constant than would have been expected and as discussed above is due to the presence of the 2,4 dichlorophenyl moiety π-bonding to the porphyrin ring. We would expect such interactions to occur in cytochrome P_450_ in addition to any bonding to the surrounding protein and are evidence that triazole moieties containing additional groups that can interact with the {Fe(PPIX)] may still be useful as therapeutic agents.

It has been found that 1-[Benzofuran-2-yl-(4-alkyl/aryl-phenyl)-methyl]-1H-triazoles; CYP26A1; is expressed in the liver, heart, pituitary gland, adrenal gland, testis, brain and placenta, of human beings [91]. The main role of CYP26A1 is thought to be homeostatic, regulating steady state levels of intracellular all-trans retinoic acid; ATRA; via a negative feedback loop [92]. The enzyme possibly has a role as a regulator of differentiation and is a possible modulator of disease states indirectly by controlling ATRA and other retinoid concentrations [93]. A number of inhibitors of ATRA metabolism have been developed [94–97] including the imidazoles and related compounds. This has been followed by studies [98] on a series of 4-alkyl/aryl benzofuran phenyl triazole derivatives has been prepared with the ethyl and phenyl derivatives shown to possess comparable inhibitory activity with that of the known CYP26 inhibitor liarozole.

Recently three series of azole piperazine derivatives that mimic dicyclotyrosine, the natural substrate of the essential Mycobacterium tuberculosis cytochrome P450, have been reported and evaluated for binding affinity and inhibitory activity against M. tuberculosis [99]. One of these series of compounds manifested some affinity for the Fe(III) atom and crystal structures of two compounds from the series show the imidazole groups positioned directly above the haem iron with binding between the haem iron and imidazole nitrogen of both compounds at a distance of 2.2 Å. We note the authors stated that although the crystal structures suggested bonding from the Fe(III) atom to the imidazole, their binding studies did not allow confirmation of this [99]. Although in this work we have only studied binding to the Fe(II) oxidation state we have previously shown that in frozen solution the orientation of imidazole groups bound in [Fe(III)(PPIX)L_2_]^+^ is pH dependent indicating that hydrogen bonding from solvent water can influence the affinity of the imidazole to the Fe(III) atom [39, 40]. The haem iron distance of 2.2 Å [99] is longer than those for both Fe(II) as discussed above (values around 2.00(2) Å [82–84] and also for those of Fe(III), we found a value of 1.975(2) Å on the structure of [Fe(III)(TPP)(4-methylimidazole)_2_]Cl [100]; however in a protein where the resolution is around 1.5 Å it is not possible to distinguish Fe(II) from Fe(III) without supporting evidence. It is likely that imidazole and indeed triazole ligated drugs could bind the P_450_ haem centre when the iron is in the reduced state as well as the low-spin Fe(III) state and thus then manifest its potential as an inhibitor. Indeed, for total inhibition the Fe(II) state must be blocked as otherwise electron reduction would affect the inhibiting molecule.

From the work we report herein it appears that for useful binding properties to the [{Fe(PPIX)] in cytochrome P_450_ the binding constant values need to be higher than 4, and the Mossbauer Δ*E*_Q_ values should be as close to 0.9 mm s^−1^ as possible.

## Conclusions

The major conclusions from the work and discussion presented in this study is the yield of many new insights into the unique chemistry of low-spin [Fe(PPIX)L_2_] complexes (where L = two identical nitrogenous ligands) and related complexes of biological significance. This is the result of the fruitful and innovative combination of the analysis of pKa—logβ_2_ data (presented in Fig. 2) with the analysis of Mössbauer parameter (Δ*E*_Q_) and the logβ_2_ data in Fig. 3. This way of combining data has already been described by us in two earlier papers [43, 44]; however, the new data presented in this work in combination with some of the results from our previous work has facilitated a more thorough presentation of this analysis method.

From this analysis some general conclusions can be made. The overall binding constant, *β*_2_ (for each ligand) is clearly related either to a property of the free ligand, here the p*K*_a_ value (Fig. 2), or to a property of the iron in the haem, here the Δ*E*_Q_, value (Fig. 3).

In Fig. 2 the different kinds of axial ligand are seen to be grouped into their structural classes. The pyridines and other aromatic five and six-membered ring ligands are well separated from the aliphatic amines, immediately indicating the difference between ligands that have both σ- and π- bonding properties and those that are purely σ-bonding. Six membered ring ligands (compounds 1–4 of Table 1) which are at the left hand end of the pyridine ligands all have lower p*K*_a_ values than most of the pyridine ligands, but their binding constants are not low indicating that the other structural properties (such as the electron donating properties of their substituents) of these ligands aid their binding to [Fe(PPIX)].

It is apparent that where steric constraints do not greatly hinder binding (e.g. the aliphatic amines, the pyridines and the imidazoles) the resulting trend within each group is that the higher the p*K*_a_ of the amine, the higher is the binding constant. Thus, increasing the affinity of a compound for protons increases its affinity for iron presumably because each bears a positive charge.

From Fig. 3 the relationship between the log *β*_2_ and the Δ*E*_Q_ value of the ligands in the [Fe(II)(PPIX)L_2_] complexes can be appreciated. The ΔE_Q_ values provide an estimate of the electron imbalance between the four nitrogen atoms of the porphyrin on the one hand and the two nitrogen atoms of the axial ligands on the other. The major factor which affects the Δ*E*_Q_ values is the distance of the axial nitrogen ligands to the iron. From Fig. 3 it was possible to group the complexes into three classes. These are:-

1. The aliphatic amines which are not sterically hindered.
2. The five-membered ring ligands.
3. The six-membered ring ligands. These split into two groups:- (a) the pyridine type ligands and the two pyrazine ligands; (b) The other four six-membered rings that have lower log β_2_ values.

We have noted that crystal structures of six coordinate low-spin iron(II) porphyrin complexes, in which the axial ligands are substituted imidazole molecule [81–84] have small Fe–N bond distances to the imidazole molecules. These small distances support our earlier findings [43, 44] and those of this work, and it follows on from this that such distances are similar to those found in naturally occurring haem proteins in which histidine is the axial ligand to the iron porphyrins.

A notable finding that we previously reported for the pyridine and aliphatic ligands and now most of the nitrogen ligands studied herein, irrespective of whether the ligand is saturated or unsaturated (in its internal bonding) and irrespective of its log *β*_2_ value, is that the Hill coefficient is significantly greater than unity. As we have discussed in this work, this finding of cooperative nitrogen binding (*h* > 1) may have implications in understanding the biological role of nitrogen ligands in haem proteins. In these proteins a nitrogen ligand is almost invariably one of the axial ligands and in the case of the electron transfer proteins two axial nitrogen ligand ligands are present.

In addition, we have shown how the results for the nitrogenous ligand binding herein are relevant to and aid the understanding of contemporary studies on the binding of inhibitor molecules on cytochrome P_450_ mono-oxygenases for therapeutic purposes.

Although the axial ligand binding studies and the complementary Mossbauer spectroscopic studies presented herein have enhanced our understanding of the low-spin [Fe(PPIX)L_2_] compounds; it is obvious that any approach that would cast further light on the bonding around the iron atoms would be beneficial. In a follow up paper to this we will consider this further.
